# Biologically realistic excitatory and inhibitory cell properties emerge from a sparse coding network

**DOI:** 10.1186/1471-2202-13-S1-P55

**Published:** 2012-07-16

**Authors:** Mengchen Zhu, Christopher J Rozell

**Affiliations:** 1 Department of Biomedical Engineering, Georgia Institute of Technology, Atlanta, GA 30332 USA; 2 Department of Electrical and Computer Engineering, Georgia Institute of Technology, Atlanta, GA 30332, USA

## 

Neurons in the primary visual cortex exhibit a baffling array of tuning properties, often unaccountable by the classical linear feedforward model. Specifically, excitatory neurons display a number of nonlinear effects collectively known as non-classical receptive field (nCRF) effects [1], and inhibitory neurons have diverse orientation tuning properties [2]. Furthermore, excitatory cells outnumber inhibitory cells by a ratio of 9:1 [3], yet the excitatory and inhibitory drives are balanced.

Efficient coding models of early vision have been shown to be able to explain key features of linear filtering properties [4] and some single cell nonlinear effects [5]. However, population statistics of nonlinear properties have not been studied in these models. In addition, inhibitory cells were not typically modeled.

Here we demonstrate that many of the aforementioned excitatory cell and inhibitory cell properties emerge naturally from a network that implements sparse coding. To be specific, several nCRF effects including surround suppression, contrast invariant orientation tuning, and cross orientation suppression emerge in the excitatory cell population as a result of sparse coding strategy; the excitatory to inhibitory cell ratio could be understood largely as a result of the overcompletness of representation; moreover, a subpopulation of inhibitory interneurons exhibit orientation tuning due to sparse recurrent connections with the principal cells; another subpopulation displays untuned properties due to low rank connectivity patterns. We also demonstrate that the network exhibits balanced excitation and inhibition, as a result of the receptive field structure.

We simulated a population of 2048 excitatory neurons with graded response described by the dynamics of locally competitive algorithm (LCA; [6]), which converges to the sparse coding representation at steady state. Inhibitory interneurons were described by linear units. The low rank and sparse recurrent connectivity pattern was a result of low rank plus sparse decomposition [7] of the LCA connectivity matrix. Non-classical receptive field effects were studied by presenting bar and drifting grating stimuli to the simulated network. Receptive fields of the inhibitory cells were mapped by sparse dots patterns.
